# A dialectical lens for AI and medical humanities: advancing responsible augmented humanism in Digital Public Health

**DOI:** 10.3389/fpubh.2026.1771351

**Published:** 2026-03-06

**Authors:** Sifan Chen, Zining Peng, Nian Liu

**Affiliations:** 1 School of Marxism, Southwest University, Chongqing, China; 2 First School of Clinical Medicine, Yunnan University of Chinese Medicine, Kunming, Yunnan, China

**Keywords:** Artificial intelligence (AI), Digital Public Health (DPH), Healthcare 5.0, intersectional AI ethics, medical humanistic values, responsible augmented humanism (RAH), Sinicized Marxist dialectics

## Abstract

Artificial intelligence (AI) creates profound dialectical tensions between technological empowerment and ethical risk in healthcare, challenging the humanistic core of medicine while offering new tools for equity. This thematic mini-review through a dialectical lens—operationalized as Sinicized Marxist dialectics—to unpack structural contradictions in AI-healthcare integration. Unlike standard bioethics or Digital Public Health (DPH) frameworks alone, this analytical tool reveals systemic power asymmetries and inequities overlooked in existing scholarship. We further integrate the Healthcare 5.0 framework and intersectional AI ethics to move beyond abstract group-based fairness toward actionable equity. The core contribution of this review is the development of the responsible augmented humanism (RAH) framework, a human-centric model operationalized across three dimensions, AI design, medical education, and multi-stakeholder governance. RAH explicitly links humanistic values to DPH principles with measurable indicators for real-world implementation. This mini-review provides a theoretically grounded, evidence-based roadmap for aligning AI innovation with humanistic care and population health equity in the AI era.

## 1 Introduction

The essence of medicine has never been confined to the biological conquest of diseases; its core lies in caring for the value of life and understanding the meaning of patients’ suffering (1). However, Artificial intelligence (AI)—including machine learning-based clinical decision support systems and generative large language models—is rapidly penetrating the medical field, profoundly reshaping clinical practice, medical education, and doctor-patient relationships (2, 3).

In the AI-driven healthcare landscape, this study anchors its normative analysis in Marxist dialectics—a philosophical tradition that emphasizes the unity of opposites and the primacy of social equity, which resonates deeply with the Sinicization of Marxism’s focus on people-centered development. This lens allows us to move beyond a simplistic technology-versus-humanism debate and instead analyze their dynamic interaction as the foundation for understanding AI’s impact on medical practice (60). Their significance lies in guiding the reconfiguration of ethical frameworks, deepening narrative functions, and fostering value-based trust and education—ensuring holistic, empathetic patient care (4).

Notably, this dialectical synthesis of technology and humanism aligns seamlessly with Digital Public Health (DPH) goals. From a Marxist perspective that critiques structural inequities (a core concern of both Marxism and its Sinicized applications), AI’s influence must be evaluated not only by its technical efficiency but also by its capacity to bridge systemic access barriers—spanning from individual care to population health equity, scalable governance, and reducing group-specific harms (e.g., digital divides, algorithmic bias) (5). Grounded in Sinicized Marxist dialectics and the principle of integrating theory with practice, this perspective provides a framework for constructing a patient-centric, AI-augmented healthcare system that balances technological innovation with social equity (6, 7).

By applying this dialectical lens, this mini-review synthesizes existing literature to systematically explore AI’s dual role, as an empowering tool and as a source of ethical conflict—within the medical humanities. It concludes by proposing a framework for ‘responsible augmented humanism (RAH)’ arguing that such a synthesis is essential for aligning technological progress with the foundational humanistic principles of healthcare.

## 2 Literature identification and selection methodology

This study is structured as a thematic mini-review. Unlike comprehensive scoping or systematic reviews, which aim for exhaustive coverage, this review focuses on the in-depth synthesis and critical analysis of key themes at the intersection of AI and medical humanistic values. To ensure methodological transparency and rigor in our literature selection, we adopted core reporting principles from the PRISMA-ScR framework (e.g., explicit inclusion/exclusion criteria) but applied them selectively to support our thematic, rather than exhaustive, aims (8).

### 2.1 Databases, search strategy and time frame

Literature retrieval was conducted across two interdisciplinary core databases: Web of Science Core Collection (WOS CC) and PubMed, covering medicine, computer science, and humanities (9, 10). The time window was set from database inception to November 30, 2025, to ensure relevance to the research theme. The search strategy combined terms for AI (“Artificial Intelligence” OR “Machine Learning” OR “Deep Learning” OR “Neural Network*” OR “Generative AI” OR “Large Language Model*” OR “ChatGPT” OR “Algorithmic”) with terms for medical humanities (“Medical Humanities” OR “Humanistic Medicine” OR “Patient-Physician Relation*” OR “Narrative Medicine”) using Boolean operators.

### 2.2 Inclusion and exclusion criteria

Inclusion criteria were: (1) English-language peer-reviewed publications; (2) studies focusing on the intersection of AI application in medicine and medical humanities. Exclusion criteria were: (1) AI-focused medical studies without medical humanities discussion; (2) Traditional medical humanities research lacking AI-related elements; (3) Non-peer-reviewed literature and non-research papers.

### 2.3 Literature screening process

**Figure 1:**
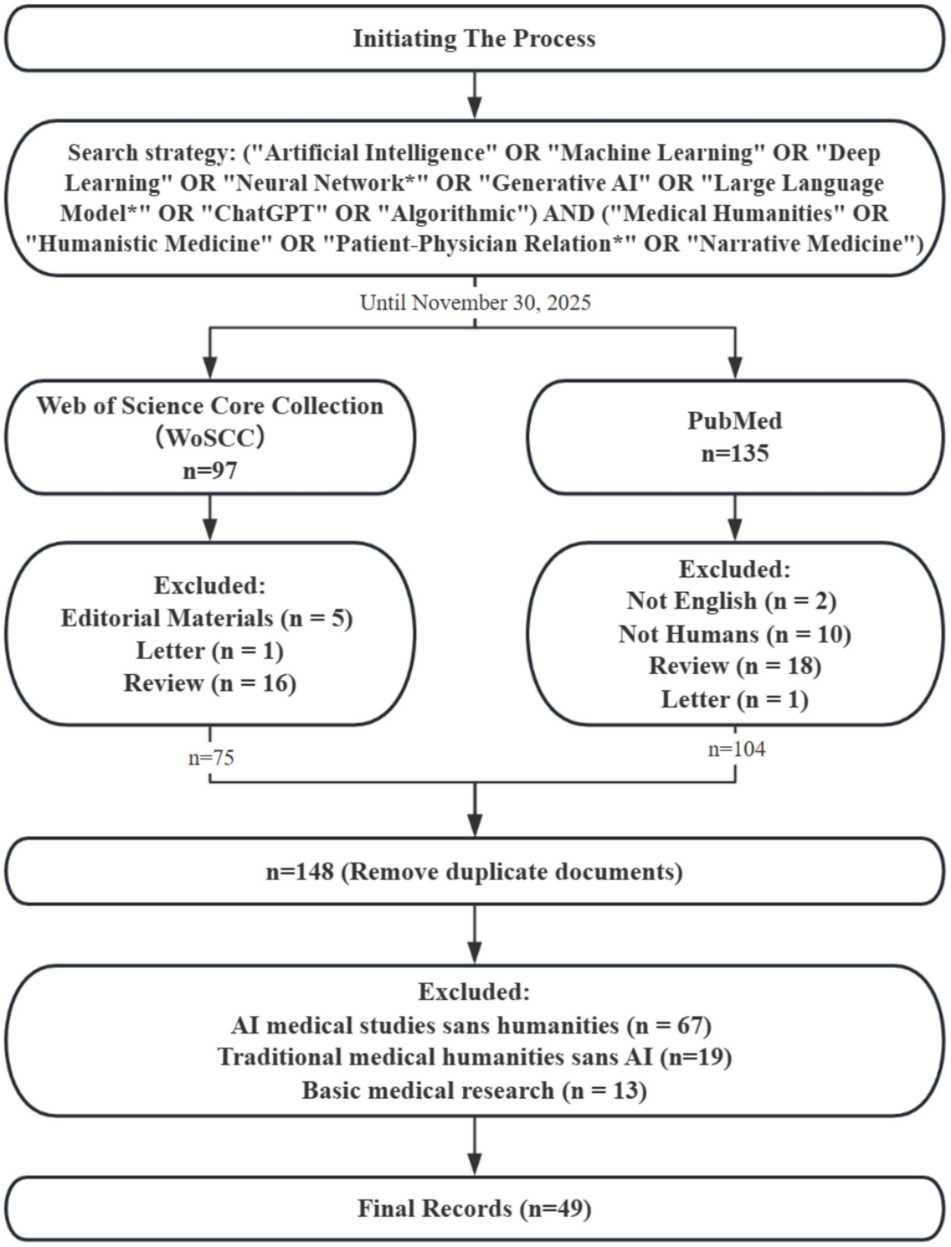
PRISMA-style flow diagram illustrating the literature screening process for this thematic mini-review. The diagram details the identification, screening, eligibility, and inclusion stages, resulting in 49 peer-reviewed articles that form the evidence base for the synthesis presented in sections. This flow is presented for transparency and does not imply a systematic or exhaustive review.

A compliant flow diagram (Figure 1) summarizes the literature selection process, identifying the final set of 49 articles that form the foundation for this mini-review’s thematic discussions.

### 2.4 Thematic synthesis and coding procedure

To ensure the rigor and transparency of the qualitative synthesis, two independent researchers (S. C. and Z. P.) conducted the coding and thematic analysis of the 49 included articles. They extracted core content on: (1) AI applications in medical humanities; (2) ethical conflicts between AI and medical humanism; and (3) proposed reconciliation strategies. These were categorized into preliminary themes and, after deliberation with a third researcher (N. L.), refined into the three core dimensions presented in this review: technological empowerment, ethical dilemmas, and reconstruction pathways. The DPH principles (equity, scalability, group harm reduction) and Sinicized Marxist dialectics (contradiction analysis) embedded as analytical lenses to ensure synthesis addressed both individual clinical and systemic population health implications of AI in healthcare.

## 3 Technological empowerment: AI as an enhancement tool for medical humanities

The integration of AI into the medical field provides new technical support for the practice of medical humanities (11). Its core value lies in reconfiguring the connotation of humanistic care by enhancing the precision of medical decision-making, optimizing educational models, and deepening doctor-patient communication. All operationalized to advance DPH’s equity and scalability goals.

### 3.1 AI-empowered humanistic care in medical education

Generative AI (e.g., large language models) demonstrates potential in simulating clinical scenarios and providing immersive learning experiences in medical education, yet empirical evidence presents a nuanced picture (11, 12). For instance, AI chatbots have been deployed to generate diverse case scenarios—including complex ethical dilemmas related to patient autonomy and empathy—to train students’ humanistic care skills, with real-time feedback mechanisms to reinforce learning outcomes (13, 14). While studies confirm that AI-assisted simulation training enhances ethical decision-making proficiency while preserving humanistic values (61). However, concerns are consistently raised that unregulated use may weaken core clinical reasoning and critical thinking, particularly in disciplines where professional judgment is paramount (15). Thus, the medical education community emphasizes the necessity of aligning its integration with evidence-based pedagogical frameworks and clear AI-learner role delineation to safeguard educational integrity, aligning with DPH’s goal of scalable, equitable humanistic education across global health systems (16).

### 3.2 Optimizing the humanistic dimensions of clinical practice

In clinical decision support, AI enhances diagnostic accuracy and reduces human error (17). Particularly in the field of rehabilitation medicine, AI generates personalized treatment plans by integrating patient data (clinical, lifestyle, and environmental—per Healthcare 5.0 principles) (18), enabling physicians to focus more on the psychological and social needs of patients (19). This represents a technology-assisted pathway toward more individualized care. However, AI performance varies substantially across health systems due to data quality, population heterogeneity and other factors, while data scarcity, inconsistent labeling and insufficient multicenter validation severely limit its generalizability and reliable deployment in resource-constrained settings (62, 63).

### 3.3 Reconstructing the trust bond of doctor-patient communication

AI tools driven by natural language processing can automatically generate plain-language health explanations, helping patients comprehend complex medical information (64). For example, when explaining the risks of genetic variations, AI platforms enhance information transparency through visual analytics, thereby mitigating the problem of information asymmetry between doctors and patients (20, 21). But their effectiveness in fostering trust is variable. Patients frequently report trust-related concerns about deficiencies in empathic engagement during AI-mediated interactions, and studies note inconsistent content quality and risks of misinterpretation, particularly among populations with low health literacy (22, 23).

### 3.4 Promoting the standardized integration of medical humanities

Current medical humanities education faces challenges such as inconsistent curricula and a lack of assessment standards (23). AI can address this by analyzing global curriculum data to design standardized humanities education modules (e.g., ethical decision-making simulations, empathy training tools) and establish quantitative assessment systems (24, 25). For instance, AI-driven feedback tools can evaluate students’ emotional expression in real-time by analyzing verbal and non-verbal cues during patient simulations, advancing DPH’s goal of scalable, high-quality humanistic education (26, 27). However, implementing these technologies faces systemic barriers, including faculty resistance, resource disparities, infrastructure deficits, and ethical guideline gaps (28). These challenges highlight the inherent tension between technological potential and the practical realities of medical education.

## 4 Ethical dilemmas: value conflicts in the tide of technology

AI’s pervasive integration in healthcare has precipitated complex ethical dilemmas rooted in structural contradictions (analyzed via Sinicized Marxist dialectics) between AI’s technological logic and medical humanism’s core values—contradictions that standard bioethics frameworks alone fail to unpack fully (29). These dilemmas challenge medical ethics principles (autonomy, justice, non-maleficence) and threaten DPH’s foundational commitments to health equity, collective well-being, and reduction of group-specific harms.

### 4.1 Autonomy, transparency and the “black box” crisis

AI-assisted diagnostic and therapeutic recommendation systems may undermine patients’ right to informed consent (a core humanistic and ethical principle) due to the “black box” nature of algorithmic decision-making: patients cannot comprehend the underlying logic of AI-generated treatment plans, rendering informed consent a formality (30). Algorithmic decisions can supplant shared decision-making, depriving patients of their right to choose and violating the ethical principle of respect for autonomy (31, 32). Compounded by AI’s non-traceable decision-making, physicians cannot explain AI diagnoses to patients—eroding the trust foundation of the doctor-patient relationship (33).

This black box crisis extends beyond clinical care to public health: AI algorithms ranking academic database search results lack transparency, with clinicians/public health researchers unable to access or influence ranking logic (34). This leads to overlooked critical literature on disease diagnosis/treatment and public health strategies, undermining the evidence-based foundation of patient care and population health initiatives (35)—a direct threat to DPH’s goal of scalable, evidence-based public health governance.

### 4.2 Algorithmic fairness, intersectionality and social justice imbalances

Algorithmic bias (rooted in non-diverse AI training data) perpetuates and solidifies medical discrimination, exacerbating health inequities—a contradiction between AI’s purported “objectivity” and the reproduction of structural social inequities (Sinicized Marxist dialectics) (33, 36). Unlike reductionist fairness frameworks that focus on single-axis identity (race/gender), intersectional AI ethics reveals that bias disproportionately harms marginalized groups with intersecting identities (e.g., low-income Black women, rural older adults) (37). With empirical evidence documenting significantly reduced accuracy of AI dermatological diagnoses for dark-skinned populations (31). AI’s black box nature further hinders bias identification, creating a cycle of harm that directly contravenes medical justice and DPH’s core principle of health equity (31). This dilemma is not merely a technical issue but a structural one: non-diverse training data reflects and amplifies existing healthcare disparities, with AI commodifying patient data in ways that reproduce power asymmetries between marginalized populations and healthcare/technology institutions (Sinicized Marxist analysis of data commodification).

### 4.3 Accountability, regulation and privacy erosion

Assigning liability for AI-related medical incidents presents a significant ethical quandary, as the boundaries of responsibility among developers, healthcare institutions, clinicians, and the algorithm itself remain ill-defined (38). When an AI system provides an erroneous surgical plan leading to patient harm, the traditional “fault-based” liability system becomes ineffective (32). More severely, existing laws lack specific provisions for AI in healthcare, resulting in regulatory lag behind technological advancement and creating significant obstacles for patients seeking legal recourse (39, 40). This regulatory vacuum is compounded by severe privacy and data security risks: medical AI relies on vast patient data sets (including genetic, lifestyle, and environmental data—Healthcare 5.0), with data collection/usage harboring high risks of breaches (18). Genetic data for AI-driven polygenic risk scores is vulnerable to misuse by insurers/employers, enabling genetic discrimination (41). Simultaneously, AI’s re-identification capacity via cross-analysis undermines traditional anonymization (42)—violating patient dignity and DPH’s mandate to protect collective population health data.

### 4.4 Core contradictions: root causes of ethical dilemmas

The empirical ethical dilemmas synthesized above stem from three core structural contradictions (Sinicized Marxist dialectics) between AI’s technological logic and medical humanism’s values—contradictions that standard bioethics/DPH frameworks alone cannot fully unpack or resolve:

1. Efficiency priority vs. Patient-centeredness: AI pursues standardization and maximal efficiency, whereas medicine must accommodate individual differences and provide emotional support (43, 44).
2. Data-driven vs. Ethical intuition: Algorithms rely on statistical patterns, while clinical decision-making often requires nuanced ethical deliberation, such as in end-of-life care (45, 46).
3. Technological iteration vs. Institutional lag: The pace of AI development far outstrips the update cycles of ethical guidelines and legal frameworks (39, 40).

These contradictions are not isolated to individual patients but threaten DPH’s foundational commitments to health equity and collective well-being, demanding system-level (not piecemeal) solutions that embed humanistic and intersectional values into AI design, education, and governance, the core focus of the novel RAH framework.

## 5 Pathways to reconstruction: toward RAH

Against the backdrop of the rapid integration of AI into medicine, medical humanistic values face an urgent need for reconstruction (47). The central challenge of preserving core humanistic elements (empathy, communication, patient-centeredness) in AI-driven innovation while leveraging technology as an empowering tool (defined as AI that enhances—not replaces—core human responsibilities in medicine) (48). RAH denotes a synergistic, human-centric framework that leverages AI’s technical capacities to enhance medical humanistic care without substituting core human responsibilities (49, 50). RAH embeds ethical principles (autonomy, justice, benevolence), Sinicized Marxist dialectics (contradiction resolution), DPH principles (equity, scalability, group harm reduction), Healthcare 5.0 (broader determinants of health), and intersectional AI ethics (explainability, transdisciplinarity) into three core, operationalizable dimensions: AI design, medical education, and multi-stakeholder governance (11, 51). This framework positions AI as a complementary tool, augmenting efficiency and accessibility, while upholding the interpersonal, value-based core of medicine that only humans can deliver.

### 5.1 Integrating humanistic values into AI design and deployment

RAH mandates the embedding of humanistic, ethical, and intersectional principles into AI design from inception (a proactive solution to the technological iteration vs. institutional lag contradiction) to avert dehumanization risks. AI algorithms trained on vast narrative datasets can support narrative and humanistic values. Yet responsible use requires developers to center narrative medicine frameworks to evaluate AI’s impact on patient experiences and align technological tools with the values of all stakeholders (patients, clinicians, marginalized communities—addressing power asymmetries via Sinicized Marxist analysis). Core DPH-aligned design principles for RAH include:

1. Algorithmic inclusivity and intersectionality: designing AI with diverse, intersectional training data (including lifestyle, environmental, and structural determinants of health—Healthcare 5.0) to prevent data bias and racial/gender/intersectional discrimination, directly addressing the algorithmic fairness dilemma (18, 47, 48).
2. Explainable AI (XAI): mandating transparent, interpretable AI models to resolve the black box crisis, enable informed consent, and allow physicians to explain AI decisions to patients—addressing autonomy/transparency dilemmas (37, 47).
3. Ethical data boundary definition: leveraging medical humanities research to delineate clinical vs. health data boundaries, preventing privacy breaches and genetic discrimination, and protecting patient dignity—addressing privacy/accountability dilemmas (47).

This integrative design approach reinforces AI’s role as an augmenter of humanism and addresses the enduring need for patient-centered, equity-focused care in the AI era, with all design choices explicitly linked to DPH principles.

### 5.2 Enhancing medical education and training

Medical education is the central arena for RAH implementation, requiring the integration of ethical AI literacy, dialectical thinking, DPH principles, Healthcare 5.0, and intersectional AI ethics into curricula to cultivate comprehensive competencies in the next generation of healthcare professionals. This restructuring addresses the standardization deficit in current medical humanities and mitigates clinician burnout and empathy decline by embedding narrative medicine and ethics (23, 52). Core DPH-aligned educational strategies for RAH include:

1. AI ethics literacy: utilizing case-based learning (e.g., generative AI in decision-making) and intersectional harm analysis to evaluate AI’s ethical constraints, ensuring technological alignment with narrative practice (27).
2. Dialectical thinking training: applying Sinicized Marxist dialectics to resolve structural contradictions in AI-healthcare integration (e.g., efficiency vs. patient-centeredness), a competency absent in standard bioethics.
3. Technological fluency + humanistic values integration: fostering algorithmic transparency/accountability and framing AI as a complement to—not a replacement for—human interaction to prevent over-reliance and hyper-mentalization (53, 54).
4. Healthcare 5.0 & DPH Competency: Training students to synthesize lifestyle, environmental, and structural determinants of health within AI-augmented care, balancing individual empathy with population health equity.

This educational framework strengthens humanistic competencies (communication, empathy) and upholds patient dignity. All strategies explicitly operationalize DPH principles: literacy programs ensure scalable governance, dialectical thinking advances equity, and Healthcare 5.0 training facilitates group harm reduction.

### 5.3 Establishing governance and policy frameworks

RAH requires institutional and systemic governance support (a solution to the accountability/regulatory vacuum contradiction) to ensure AI’s ethical integration and promote multi-stakeholder participation—core DPH and Sinicized Marxist principles of collective agency and inclusive governance. RAH mandates inclusive governance frameworks that address data privacy, algorithmic bias, and healthcare injustice (55), with core DPH-aligned governance strategies (explicitly linked to equity, scalability, group harm reduction) including:

1. Transparency and accountability regulations: mandating XAI reporting and bias audits to mitigate technological alienation and reinforce medical humanities (DPH link: scalable governance);
2. Multi-stakeholder and transdisciplinary collaboration: institutionalizing transdisciplinary dialogues (clinicians, technologists, marginalized communities) utilizing narrative medicine to assess social impacts (DPH link: equity and collective agency);
3. Dynamic ethical review mechanisms: establishing adaptive review boards to bridge the technological iteration vs. institutional lag contradiction, prioritizing Healthcare 5.0 determinants and intersectional harm analysis (DPH link: group harm reduction);
4. Global DPH governance collaboration: advancing equity-centered international regulations to prevent cross-border harm and treat healthcare as a public good (DPH link: global scalability).

This framework shifts medical humanities from an anthropocentric paradigm to an inclusive, augmented practice, positioning AI as a catalyst for restoring humanity in care and reinforcing the centrality of healthcare professionals.

### 5.4 Conclusion: operationalizing RAH

Operationalizing RAH requires synergizing technological empowerment with ethical governance via integrated design, education, and policy frameworks. This approach embeds core DPH tenets—scalable population-level oversight, equity-centered innovation, and inclusive participation—while resolving structural contradictions through Sinicized Marxist dialectics. RAH redefines medical humanism not as a static legacy, but as a dynamically augmented practice where empathy and justice are continuously reaffirmed. A brief summary is shown in Table 1.

**Table 1:** Operationalized components of RAH in healthcare with explicit DPH principle links.

AI use case	Humanistic value at stake	Risk mechanism	Mitigation lever (design/education/governance)	Explicit DPH principle link	Measurable indicator (real-world assessment)
Diagnostic assistance	Patient autonomy & trust	Misdiagnosis due to algorithmic/intersectional bias	Design: diverse, intersectional training data (lifestyle/environmental—Healthcare 5.0); governance: mandatory bias audits and ethical review boards	Equity/group harm reduction	1. Diagnostic accuracy rate across intersectional demographic groups; 2. Algorithmic bias audit scores; 3. Patient informed consent comprehension rate
Personalized treatment planning	Beneficence & non-maleficence	Over-reliance on AI recommendations; exclusion of broader health determinants (Healthcare 5.0)	Education: critical thinking in AI use; design: XAI models	Scalability/equity	1. Patient clinical outcomes across demographic groups; 2. Treatment adherence rate; 3. Clinician AI over-reliance score
Medical education Augmentation	Empathy & professional development	Decreased face-to-face interaction skills; lack of intersectional AI ethics literacy	Education: blended learning with human mentorship + intersectional AI ethics modules; design: simulation-based training with diverse, intersectional patient scenarios	Scalability/Group Harm Reduction	1. Student empathy assessment scores; 2. Student intersectional AI ethics competency score; 3. Student satisfaction with human-AI blended learning
Patient-doctor communication support	Trust & relationship building	Information overload or misinterpretation; low health literacy disparities	Design: plain-language health explanations; governance: patient feedback loops	Equity/group harm reduction	1. Patient satisfaction score across health literacy/demographic groups; 2. Patient health information comprehension rate; 3. Doctor-patient relationship trust score
Clinical decision support systems	Justice & equity	Disparities in AI access or application; exclusion of structural health determinants	Governance: Policy frameworks for equitable AI deployment; design: inclusive design principles	Equity/scalability	1. AI utilization rate across demographic/geographic/socioeconomic groups; 2. AI access gap score; 3. Structural determinant integration rate in AI decision-making

## 6 Discussion

The reconstruction of medical humanistic values in the AI era is shaped by the structural contradictions between AI’s technological empowerment and ethical dilemmas—contradictions that are best analyzed and resolved via a synergistic framework of Sinicized Marxist dialectics and DPH (50, 56). This dialectical-DPH framework reveals three critical, underaddressed research gaps in current AI-medical humanities scholarship, which restrict the holistic application of medical humanism in AI-driven health systems and fail to center Healthcare 5.0 or intersectional AI ethics (18, 37). This section elevates the study’s insights by identifying these gaps, advancing systemic, DPH-aligned solutions, and discussing the broader theoretical and practical implications of RAH for global AI healthcare governance.

### 6.1 Critical research gaps in AI-medical humanities scholarship

1. Narrow focus on clinician-patient interactions: most studies overlook structural inequities, such as the digital divide (e.g., limited AI access for low-income or older adult populations) and algorithmic bias that amplifies subgroup harms (e.g., racial/gender disparities in diagnostic accuracy) (36, 55). This oversight ignores health equity as an inherent component of humanistic care and fails to integrate Healthcare 5.0’s broader determinants of health—contradicting both Sinicized Marxist principles of social equity and DPH’s population health goals.
2. Insufficient system-scale governance: regulatory frameworks prioritize individual clinical incidents over scalable accountability chains (e.g., cross-border AI data governance, health system-wide ethical review), failing to address population-level risks of AI deployment (39, 40). And lack transdisciplinary collaboration, failing to address the technological iteration vs. institutional lag contradiction and threatening DPH’s scalable governance goals
3. Overlooked group harms and stakeholder participation: group-level risks (e.g., AI-enabled health surveillance, disease stigma) and inadequate community engagement in AI design perpetuate power imbalances, contradicting DPH’s emphasis on collective agency and inclusive health systems (57, 58). And lacks meaningful community/marginalized stakeholder engagement in AI design—perpetuating power asymmetries (Sinicized Marxist analysis) and contradicting DPH’s emphasis on collective agency and inclusive health systems.

### 6.2 Systemic, DPH-aligned solutions to advance RAH and medical humanistic reconstruction

Addressing the above research gaps requires a multi-dimensional, transdisciplinary approach that integrates DPH principles, Healthcare 5.0, intersectional AI ethics, and Sinicized Marxist dialectics—extending the RAH framework to systemic and global levels. These novel, actionable solutions are grounded in the study’s empirical synthesis and theoretical framework:

1. Educational reform: extend RAH’s medical education strategies to integrate DPH-focused population health training and Healthcare 5.0 literacy into all medical curricula, equipping clinicians with a dual lens of individual empathy and population health equity (36). Modular teaching on algorithmic transparency and health equity will ensure alignment with humanistic care goals.
2. Targeted, interdisciplinary empirical research: priority should be given to interdisciplinary empirical studies exploring: AI-driven solutions for reducing health disparities; scalable governance models; and community-co-designed AI tools to avoid subgroup harms (59). Develop a unified ethical evaluation framework for humanistic AI that integrates biomedical ethics, DPH principles, Healthcare 5.0, and intersectional AI ethics—strengthening the evidence base for RAH implementation.
3. Global, equity-centered policy and governance: international collaboration should drive dynamic, equity-centered regulations and multi-stakeholder governance. Policies must prioritize healthcare as a public good, ensuring AI innovations reinforce rather than undermine medical humanistic values (65). Mandate DPH impact assessments for all AI healthcare tools, with criteria including intersectional harm analysis, Healthcare 5.0 data integration, and scalability for resource-constrained settings.

These solutions address the structural contradictions and research gaps identified in this study, and position RAH as a scalable, adaptable framework for AI healthcare governance across global health systems.

### 6.3 Broader theoretical and practical implications

The dialectical-DPH-RAH framework yields two primary theoretical contributions: it operationalizes Sinicized Marxist dialectics as a pragmatic tool for resolving structural contradictions in AI-healthcare integration, and it redefines medical humanism by embedding Healthcare 5.0 and intersectional AI ethics to align individual care with DPH population goals. Practically, the framework provides actionable guidance for institutions and developers. By centering intersectional harm analysis and stakeholder participation, RAH ensures AI innovation advances health equity and patient dignity, particularly for marginalized communities.

### 6.4 Final conclusion

Reconstructing medical humanism in the AI era necessitates extending beyond individual interactions to embrace DPH principles of equity, scalability, and collective well-being, centered on Healthcare 5.0 determinants and intersectional AI ethics. This study introduces the RAH framework, grounded in Sinicized Marxist dialectics, to resolve contradictions between technological logic and humanistic values. By integrating these principles into design, education, and governance, RAH ensures AI acts as a catalyst for revitalizing—not replacing—the human core of medicine. Future research must focus on empirical validation across diverse global health systems and long-term assessment of RAH’s impact on equity, confirming that medical humanism is a dynamically augmented practice requiring the continuous reaffirmation of empathy and justice.
